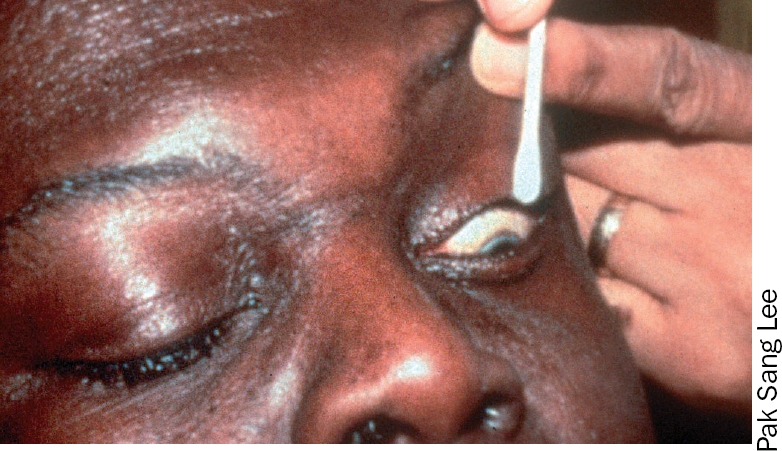# How to clean eyelids

**Published:** 2011-09

**Authors:** Sue Stevens

**Affiliations:** Former Nurse Advisor, Community Eye Health Journal, International Centre for Eye Health, London School of Hygiene and Tropical Medicine, Keppel Street, London WC1E 7HT, UK

**Figure:**
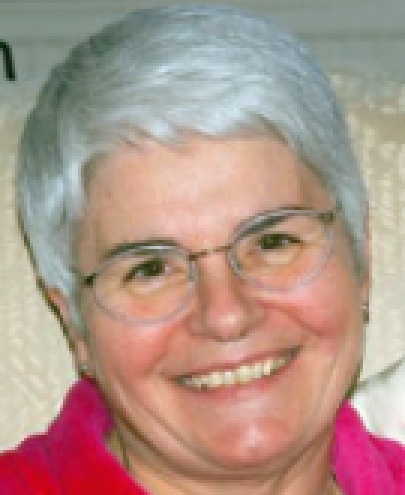


## Before performing an eye procedure

- **Wash your hands** (and afterwards too).
- Position the patient comfortably with head supported.
- **Avoid distraction** for yourself and the patient.
- Ensure good lighting.
- Always **explain to the patient** what you are going to do.

## Reasons for cleaning eyelids

- **Basic eye hygiene**: to remove any discharge before instillation of eye drops or applying eye ointment, or before applying post-operative eye dressings.
- **Blepharitis**: to remove crusting on the eyelid margins.

## You will need

- sterile cotton buds
- sterile gauze swabs
- salt
- sodium bicarbonate (more effective than salt for blepharitis)
- teaspoon
- jug
- small sterile pot

**Figure 1:**
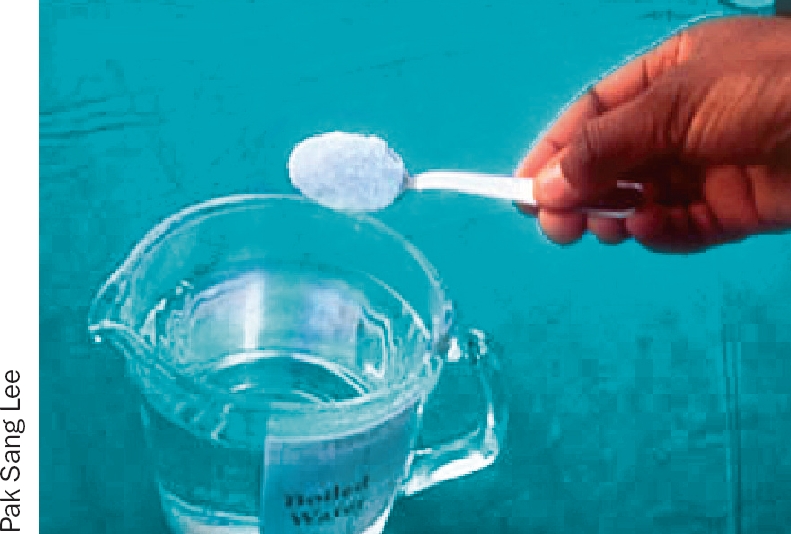


**Figure 2:**
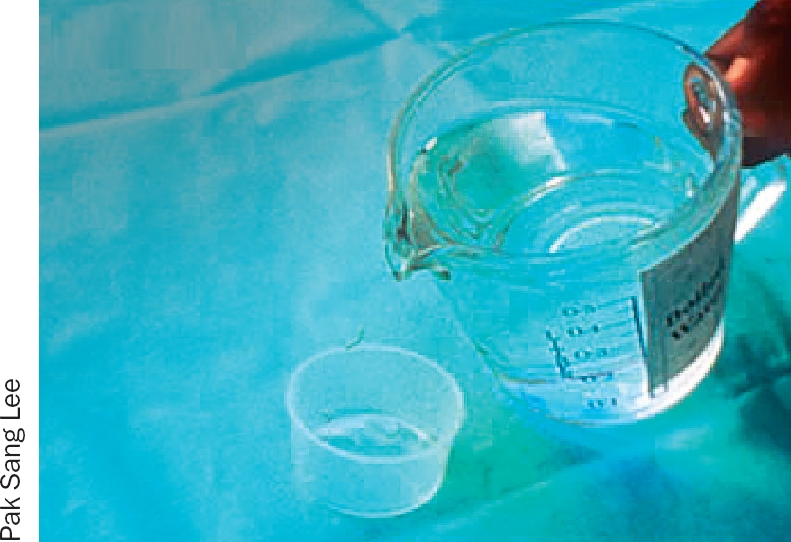


## Preparation

- Dissolve 1 heaped teaspoonful of salt or sodium bicarbonate in a jug containing 500 ml of boiled water (half a litre); allow this solution to cool (Figure 1).
- Pour a very small amount of the solution into a small sterile pot on a clean surface (Figure 2).

**Figure 3:**
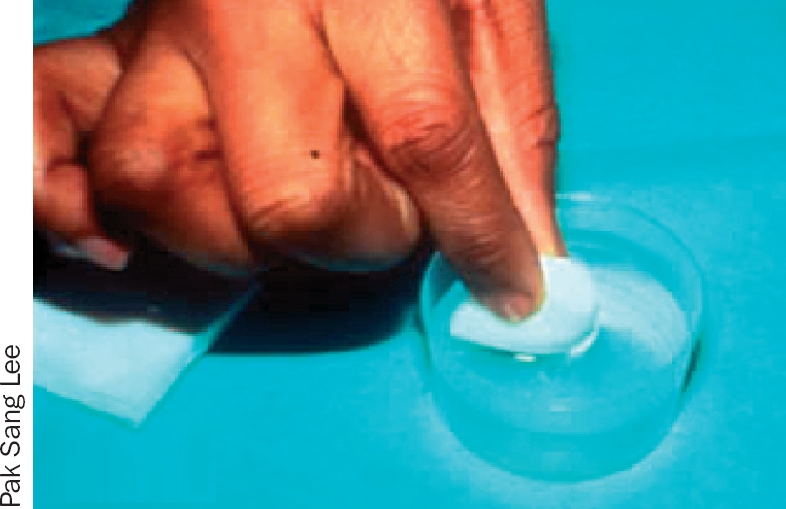


**Figure 4:**
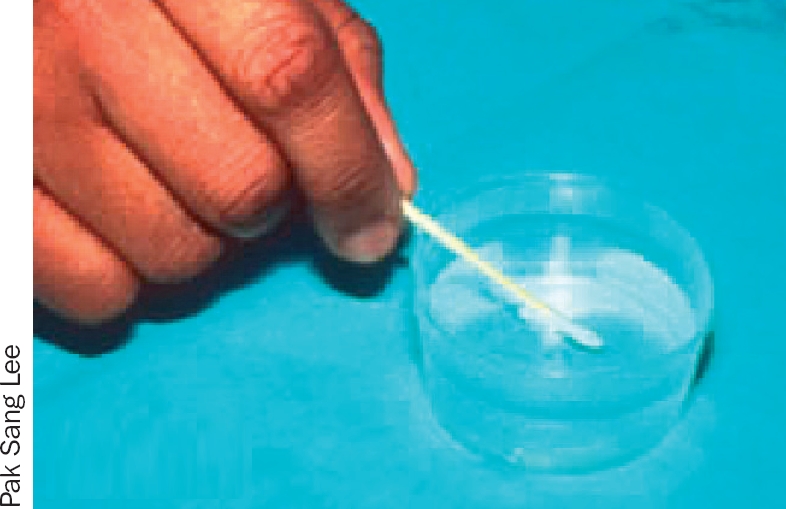


**Figure 5:**
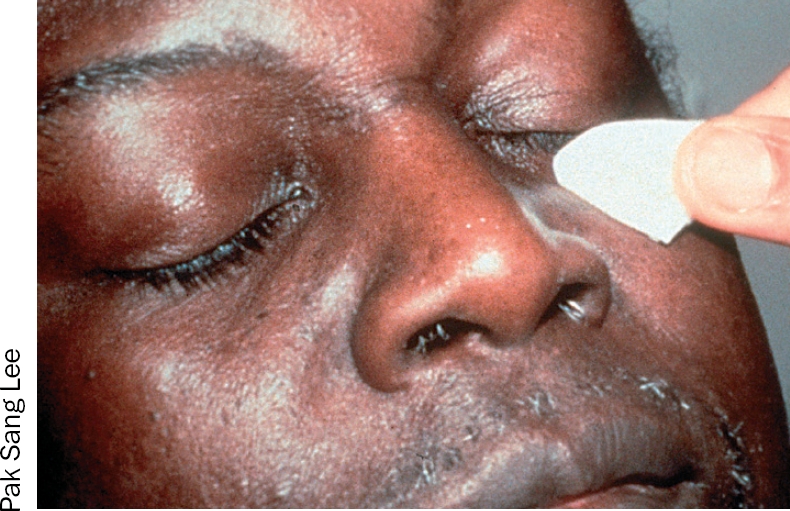


**Figure 6:**
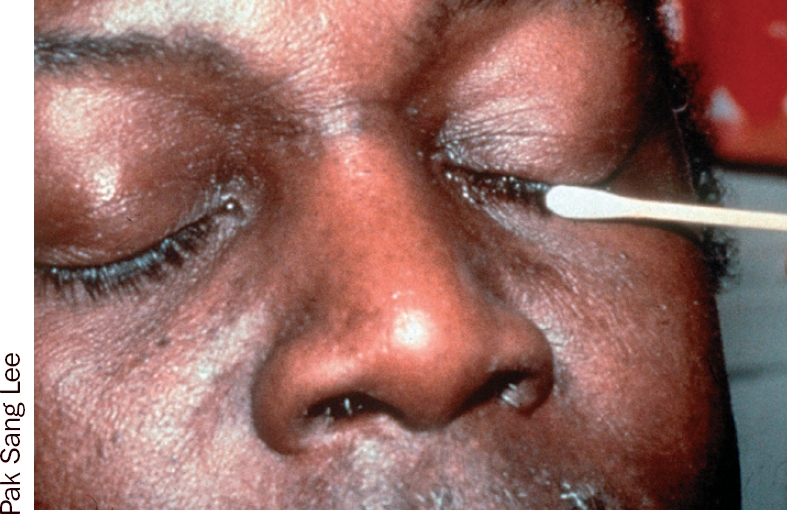


## Method

### 1 The eyelashes

- Ask the patient to close both eyes.
- Take a folded gauze swab or cotton bud.
- Moisten the swab or bud with the prepared solution (Figures 3 and 4).
- With the swab or bud, clean gently along the eyelashes in one movement, from inner to outer canthus (Figures 5 and 6).
- Discard the swab or bud after use.

### 2 The lower eyelid

**Figure 7:**
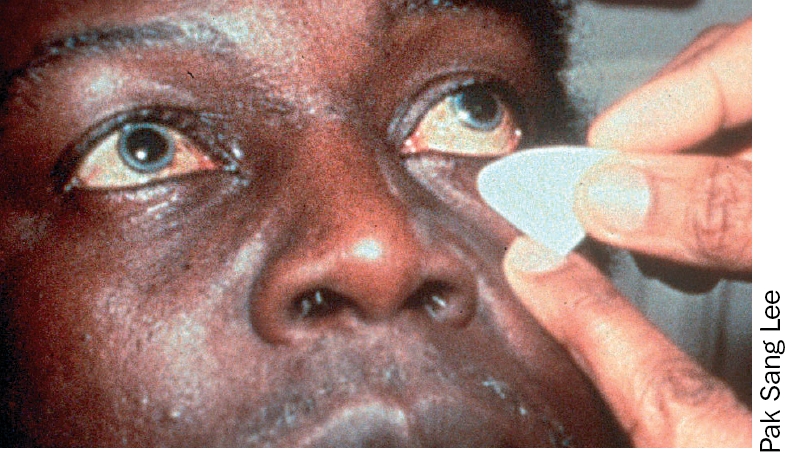


**Figure 8:**
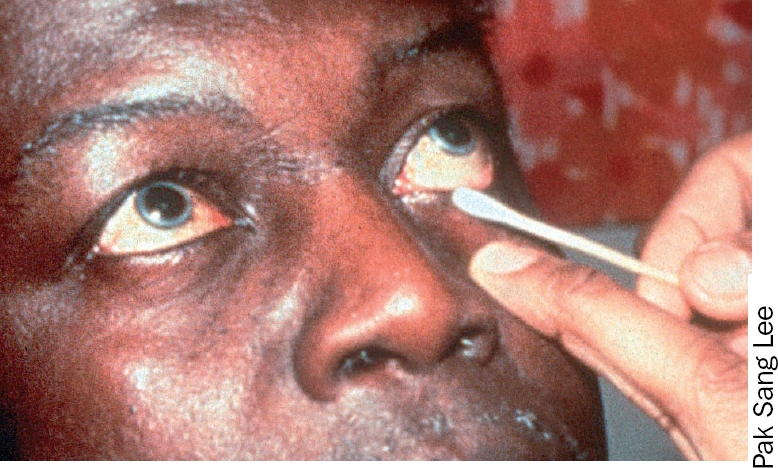


- Ask the patient to look up.
- With one hand, take a new swab or bud and moisten it in the solution.
- With the index finger of the other hand, gently hold down the lower eyelid.
- With the swab or bud, clean gently along the lower eyelid margin in one movement from inner to outer canthus (Figures 7 and 8).
- Discard the swab or bud after use.

### 3 The upper eyelid

**Note**: extra care is needed when cleaning the upper eyelid margin. Try to keep the cornea in view throughout and avoid touching it with the swab or bud.

**Figure 9:**
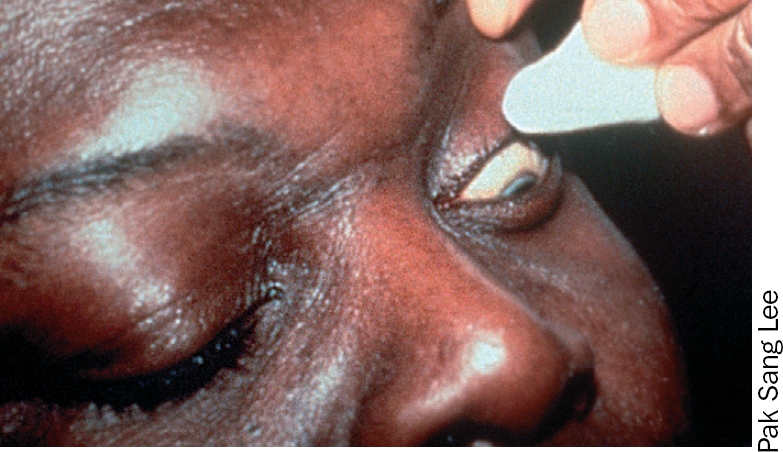


- Ask the patient to look down.
- With one hand, take a new swab or bud and moisten it in the solution.
- With a thumb or finger of the other hand, gently ease the upper eyelid up against the orbital rim (just below the eyebrow), taking care not to put any pressure on the eyeball.
- With the swab or bud, clean gently along the upper eyelid margin in one movement from inner to outer canthus (Figures 9 and 10).
- Discard the swab or bud after use.

**Note: always use a new swab or bud each time**

- If the eyelids are very sticky or encrusted, it will be necessary to repeat any part of the above procedure until all debris or discharge is removed.
- Finally, discard the unused remainder of the solution.

**Figure 10:**